# Pupillometry as a Novel Tool for Pain Monitoring: Evaluating the Antinociceptive Effect of Intravenous Lidocaine During Orotracheal Intubation

**DOI:** 10.3390/jcm15051840

**Published:** 2026-02-28

**Authors:** Małgorzata Braczkowska-Skibińska, Ewa Lepiarczyk, Lidia Glinka, Iwona Piotrowicz, Paweł Radkowski, Ewa Mayzner-Zawadzka, Marta Majewska

**Affiliations:** 1 Department of Anaesthesiology and Intensive Care, Faculty of Medicine, Collegium Medicum, University of Warmia and Mazury in Olsztyn, 10-082 Olsztyn, Poland; malgorzata.braczkowska@uwm.edu.pl (M.B.-S.); iwona.piotrowicz@uwm.edu.pl (I.P.); pawel.radkowski@uwm.edu.pl (P.R.); ewa.mayzner@gmail.com (E.M.-Z.); 2 Department of Human Physiology and Pathophysiology, Faculty of Medicine, Collegium Medicum, University of Warmia and Mazury in Olsztyn, 10-082 Olsztyn, Poland; ewa.lepiarczyk@uwm.edu.pl; 3 Hospital in Ostróda, 14-100 Ostróda, Poland; lidka.glinka@gmail.com

**Keywords:** pupillometry, pupillary dilation reflex (PDR), Pupillary Pain Index (PPI), nociception monitoring, orotracheal intubation

## Abstract

**Background/Objectives**: Pupillometry offers a non-invasive method for assessing nociceptive responses during anesthesia. This study aimed to evaluate the effects of intravenous lidocaine on pupillary reflex dilation (PDR) and the Pupillary Pain Index (PPI) during general anesthesia with orotracheal intubation. **Methods**: In this prospective, randomized, single-blind trial, 90 ASA I–II patients aged 18–65 years, scheduled for elective surgery under general anesthesia, were enrolled. Participants were randomized into three groups: control, study (lidocaine 1.5 mg/kg), and placebo. Standardized anesthesia induction was performed using propofol, fentanyl, and rocuronium. Pupil diameter was measured using the Algiscan pupillometer. PDR was assessed during intubation, while PPI was measured five minutes post-intubation through controlled electrical stimulation. Hemodynamic parameters and BIS values were recorded throughout. Eighty-six patients completed the study. No significant differences in demographics, anesthetic drug doses, or hemodynamic parameters were noted between groups. **Results**: PDR during intubation showed no significant difference between the control and study groups (median dilation: 0.34 mm vs. 0.33 mm; *p* = 0.76), but was significantly lower in the lidocaine group compared to placebo (median dilation: 0.33 mm vs. 0.50 mm; *p* = 0.02). PPI scores did not differ significantly between groups (*p* > 0.05). A positive correlation was observed between PDR and BIS values, indicating that lighter anesthesia depth increased PDR response. No such correlation was found with PPI. **Conclusions**: Intravenous lidocaine attenuates the pupillary response to nociceptive stimuli during orotracheal intubation but does not influence PPI scores. Pupillometry remains a valuable adjunct for intraoperative nociceptive monitoring.

## 1. Introduction

Direct laryngoscopy and orotracheal intubation pose a significant risk of adverse hemodynamic alterations. These changes are primarily mediated by sympathetic nervous system activation, triggered by mechanical stimulation of the upper airway. The physiological manifestations include hypertension and tachycardia. In susceptible individuals, particularly those with pre-existing cardiovascular or cerebrovascular pathologies, these hemodynamic disturbances can precipitate severe, potentially life-threatening events such as cardiac arrhythmias, myocardial infarction, or cerebrovascular accidents [1]. Consequently, strategies aimed at mitigating or preventing such hemodynamic instability during tracheal intubation are crucial for patient safety. Precise monitoring of the nociceptive–antinociceptive balance should serve as the primary guide for the titration of analgesic drugs [2]. Evaluating the efficacy of analgesia presents a significant challenge in anesthesiology. Current perioperative assessment of the analgesia component largely relies on non-specific clinical endpoints, including patient movement, tachycardia, hypertension, lacrimation, and diaphoresis. These indicators, however, can be unreliable and may inaccurately suggest inadequate analgesia due to confounding factors such as the patient’s underlying physiological state or concurrent pharmacotherapy [3]. In recent years, the medical device market has seen the introduction of various monitoring systems designed to assist clinicians by providing surrogate variables indicative of the nociception–anti-nociception balance [4]. Among these innovations, pupillometry has emerged as a particularly promising technique. Pupillary diameter is dynamically regulated by the interplay between the sympathetic nervous system, which induces mydriasis, and the parasympathetic nervous system, which elicits miosis [5]. The pupillary dilation reflex (PDR) has demonstrated utility in quantifying the analgesic component within a balanced anesthetic regimen [6]. This observation led to the hypothesis that PDR could serve as a reliable objective index of pain intensity, thereby guiding the administration of morphine in the immediate postoperative period [7].

This prospective experimental study introduces pupillometry with automatic pupillary dilation reflex (PDR) assessment as an objective method for evaluating the adequacy of analgesia during orotracheal intubation. Pupillometry has been previously investigated as a tool for assessing nociception and adequacy of anesthesia in various surgical settings, including endoscopic sinus surgery, where pupillary reflex parameters were shown to correlate with intraoperative analgesic requirements [8]. However, its specific application during orotracheal intubation, a short-lasting but highly intense nociceptive stimulus, has not been extensively characterized, particularly in the context of intravenous lidocaine administration.

This study addresses this important gap by hypothesizing that PDR could serve as a reliable and objective index of pain intensity, potentially guiding morphine titration in the immediate postoperative period. The primary objective was to determine the clinical usefulness of pupillometry in evaluating analgesia levels during orotracheal intubation, while also assessing the efficacy of intravenous lidocaine in modulating both the pupillary and hemodynamic responses to this noxious event. Secondary endpoints included exploring the correlation between the depth of anesthesia, as measured by the Bispectral Index (BIS), and pupillary reactivity, specifically changes in PDR and the Pupillary Pain Index (PPI).

## 2. Materials and Methods

### 2.1. Study Design and Participants

**Figure 1:**
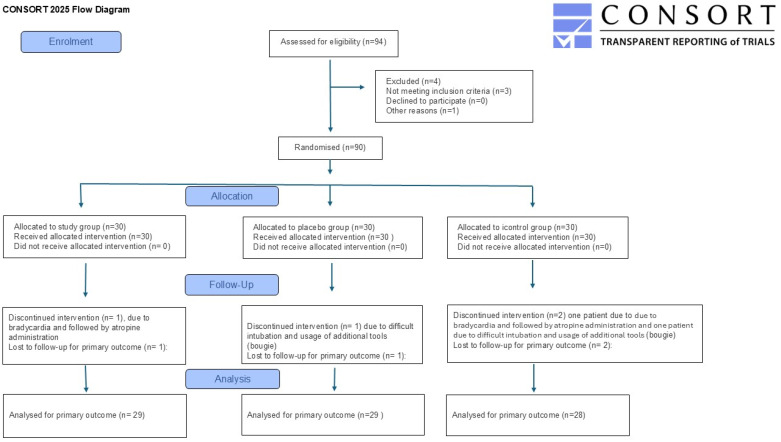
Flow of participants through the randomized controlled trial.

This study was a single-blind, randomized, prospective investigation approved by the Bioethics Committee of the University of Warmia and Mazury on 21 February 2019 (approval number 11/2019). The trial was registered at ClinicalTrials.gov (ID: NCT04000126; 25 June 2019). A total of 90 participants were initially recruited, aged 18 to 65 years, with an American Society of Anesthesiologists (ASA) physical status classification of I or II and a body mass index (BMI) below 30 kg/m^2^. All participants were scheduled for elective surgical procedures requiring general anesthesia with orotracheal intubation. Written informed consent was obtained from all participants prior to inclusion in the study. Patients with pre-existing ocular pathologies that could interfere with pupillometry were excluded, as were individuals with a history of alcohol, nicotine, or opioid abuse, chronic pain, or regular use of analgesic medications. The selected participants were then randomly assigned into three distinct groups: group 1 (a control group), group 2 (a study group), and group 3 (a placebo group). A preliminary sample size calculation was performed prior to study initiation, based on the expected difference in the Pupillary Pain Index (PPI) between groups, using data derived from pilot observations. The calculation assumed a two-sided alpha level of 0.05 and a statistical power of 80%, indicating that a minimum of 25 patients per group was required. To account for potential dropouts, we planned to include an additional five participants per group. Of the 90 patients enrolled, 86 completed induction of general anesthesia and were included in the intraoperative assessment, with 28 in the control group, 29 in the lidocaine group, and 29 in the placebo group. Four patients were excluded at the anesthesia stage: two from the control group, one from the study group, and one from the placebo group. One patient in the placebo group and one in the control group were excluded due to the necessity of using an intubation stylet for difficult airway management, which constituted an additional nociceptive stimulus and compromised the uniformity of the intubation technique. One patient from the control group and one from the study group were disqualified due to the need for atropine administration following bradycardia induced by intravenous anesthetic agents used during induction. Atropine, as an antagonist of the postganglionic M_1_ and M_2_ cholinergic receptors, increases heart rate and induces pupillary dilation, which could confound pupillometric measurements. The flow of participants through each stage of the study, including recruitment, allocation, and final analysis, is detailed in Figure 1.

### 2.2. Anesthetic Management and Data Acquisition

Upon arrival in the operating room, all patients underwent intravenous cannulation and were connected to standard monitoring equipment, including electrocardiography (ECG), non-invasive blood pressure (NIBP), pulse oximetry (SpO_2_), Bispectral Index (BIS), and Train-of-Four (TOF) monitoring. Baseline hemodynamic parameters, including systolic blood pressure (SBP), diastolic blood pressure (DBP), heart rate (HR), and SpO_2_, were recorded prior to anesthetic induction.

Ten minutes before induction, the study group received 1.5 mg/kg intravenous lidocaine (Lignocainum hydrochloricum WZF 1%, Polfa Warszawa S.A., Warsaw, Poland) diluted in 100 mL of 0.9% normal saline, while the placebo group received 100 mL of 0.9% normal saline. Anesthesia was then induced with propofol 2 mg/kg (Propofol 1%, MCT/LCT Fresenius Kabi 10 mg/mL), fentanyl 3 µg/kg (Fentanyl WZF, Polfa Warszawa, 50 mgc/mL), and rocuronium 0.6 mg/kg (Rocuronium Kabi, 50 mL/5 ml, Fresenius Kabi). Orotracheal intubation was performed by an experienced anesthetist using a standard Macintosh laryngoscope blade once optimal muscle relaxation (TOF = 0) and an adequate depth of anesthesia (BIS ≤ 50) had been achieved.

During intubation, the PDR protocol was initiated and simultaneous BIS and hemodynamic variables were recorded. Five minutes post-intubation, the Pupillary Pain Index (PPI) protocol was performed, with concurrent BIS measurement. Mean arterial pressure (MAP), DBP, HR, and BIS values were recorded every minute from the onset of anesthesia induction until the completion of the PPI protocol. Throughout the procedure, the BIS value was maintained at or below 50; if this target was not met, an additional intravenous bolus of propofol (1 mg/kg) was administered. All recorded complications, such as bradycardia (HR < 50 bpm) and hypotension (SBP < 90 mmHg), were also documented.

### 2.3. Pupillometry Measurement Techniques and Pain Indices

Pupillary diameter measurements were conducted using the Algiscan video pupillometer (ID Med, Marseille, France). This non-invasive device employs an infrared camera for instantaneous recognition, tracking, and measurement of the pupil. The Algiscan is CE-marked for clinical use but not FDA-approved for intraoperative nociception monitoring; therefore, its use in this study represents an investigational application. During assessment, the upper eyelid of the measured eye was opened. Optimal device positioning, consistent pupil-to-camera distance, and environmental darkness were ensured by a rubber cup placed on the orbit, avoiding direct corneal contact. The contralateral eye remained closed to minimize the consensual light response.

The PDR protocol involved a 60 s video recording to quantitatively assess pupil size during nociceptive stimulation, specifically during orotracheal intubation. Following a baseline pupil assessment (in mm), real-time measurements included pupil enlargement, maximal dilation (expressed as both a percentage and in millimeters), and the PDR index. Adequate pain control was defined as a less than 12% increase in pupil diameter from baseline.

For the PPI protocol, two adhesive electrodes were positioned on the medial aspect of the patient’s left forearm. The negative electrode was placed proximally to the wrist on the ulnar side, while the positive electrode was positioned 3 to 5 cm more proximally, also on the ulnar side. After baseline assessment, a stepwise, gradually increasing electrical stimulation was applied to the left forearm via these electrodes, which were connected to the pupillometer. This protocol yielded baseline pupil size (mm), pupillary dilation to pain (%), and the PPI. The PPI quantifies pupil dilation in response to a continuously increasing 1 s electrical stimulus, ranging from 10 to 60 mA in 10 mA increments. The stimulus ceased upon detection of a 13% increase in the PDR. The device then calculated the PPI, assigning a score from 1 (pupillary dilation < 5% with maximal stimulation intensity) to 10 (pupillary dilation > 13% with a 10 mA stimulus). Adequate pain control was defined by a PPI score of ≤4 [9].

### 2.4. Statistical Analysis

All statistical analyses were performed using TIBCO Statistica™ (version 13, 2017; https://www.tibco.com/; accessed on 20 September 2019). Descriptive statistics were used, including mean, standard deviation, median, and subgroup percentages, to summarize the data. The Shapiro–Wilk test assessed the normality of variable distribution.

Given the nature of the data, a non-parametric tests was employed. The Wilcoxon test was used to evaluate differences over time. To compare median values of parameters between subgroups (control vs. study, control vs. placebo, study vs. placebo), the Mann–Whitney U test was used. A chi-squared tests was performed to compare proportions across subgroups. Finally, linear relationships were examined between variables using Spearman’s Rank Correlation (correlation coefficient, r). A *p*-value < 0.05 was considered statistically significant.

## 3. Results

Out of 90 patients enrolled, 86 completed the trial. A propofol adjustments were made when necessary to maintain the desired depth of sedation. All pupil measurements were taken in the absence of hypoxia or hypercapnia. Importantly, patients did not receive any anti-emetic treatment or benzodiazepine premedication before pupillary analyses.

**Figure 2:**
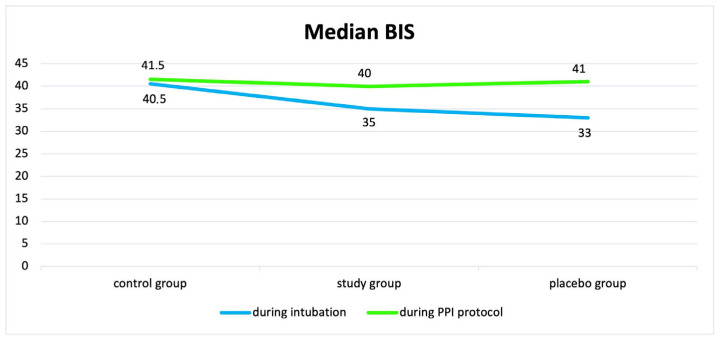
Comparison of anesthetic depth between study groups during intubation and during the Pupillary Pain Index (PPI) protocol. The graph presents a comparison of anesthetic depth, as measured by the Bispectral Index (BIS) during intubation and during the PPI protocol. Data are stratified across three patient cohorts: Group 1 (control), Group 2 (study), and Group 3 (placebo). The Pupillary Pain Index (PPI) refers to the indexed pupillary reflex to pain.

The groups showed no significant differences in demographic profiles, including age, sex, height, weight, BMI, and ASA physical status (*p* < 0.05). We also found no statistically significant differences in anesthetic drug doses between the groups (*p* < 0.05). While the depth of anesthesia, as measured by the BIS index, was statistically higher in group 1 (control group) during orotracheal intubation (BIS = 40.5), there was no difference between group 2 (study group) (BIS = 35) and group 3 (placebo group) (BIS = 33). Despite this statistical difference, all groups achieved BIS values indicative of a deep anesthetic state adequate for surgery and orotracheal intubation. There were no statistically significant differences in BIS values between groups during the PDR protocol (*p* < 0.05; Figure 2). It should be noted that no statistically significant difference in PDR was observed during tracheal intubation between group 2 (study group) (mean value: 0.33 mm) and group 1 (control group) (median value: 0.34 mm), with a *p*-value of 0.76 (Table 1).

**Figure 3:**
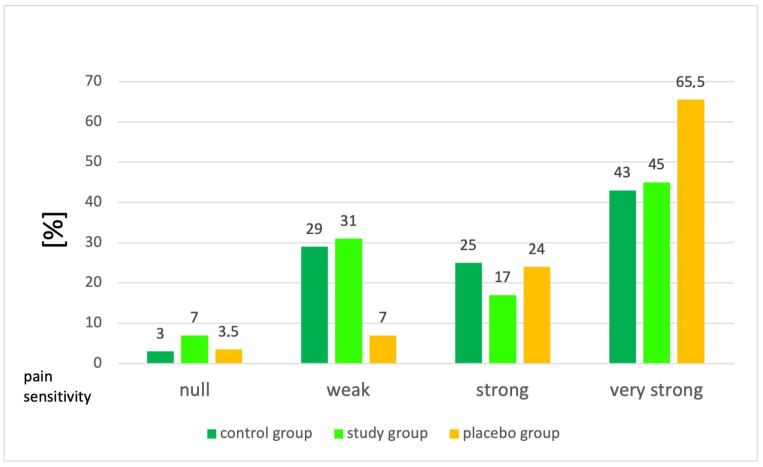
Qualitative comparison of pupillary dilation reflex (PDR) across control, study, and placebo groups. Figure illustrates the qualitative scale of the PDR, comparing patients from group 1 (control), group 2 (study), and group 3 (placebo).

A statistically significant difference in pupil dilation was observed between group 2 (study group) (median dilation: 0.33 mm) and group 3 (placebo group) (median dilation: 0.5 mm; *p* = 0.02; Table 2). The median percentage of pupil dilation was 16% in the study group, compared to 26% in the placebo group (*p* = 0.04). Based on the Pupillary Dilation Reflex (PDR) scale, patients in the study group exhibited high sensitivity to pain, whereas patients in the placebo group demonstrated very high sensitivity to pain (Figure 3).

Patients exhibiting very strong pain sensitivity constituted the largest proportion across all groups: 65.5% in the placebo group, 45% in the study group, and 43% in the control group. Conversely, weak pain sensitivity was observed in 31% of patients in the study group, a notably higher percentage compared to only 7% in the placebo group. No statistically significant differences were observed in pupillary variation values in response to nociceptive stimulation during the PPI protocol among the groups (*p* > 0.05). The mean PPI scores were 6 for the control group, 5 for the placebo group, and 6 for the study group (Table 3a–c).

**Figure 4:**
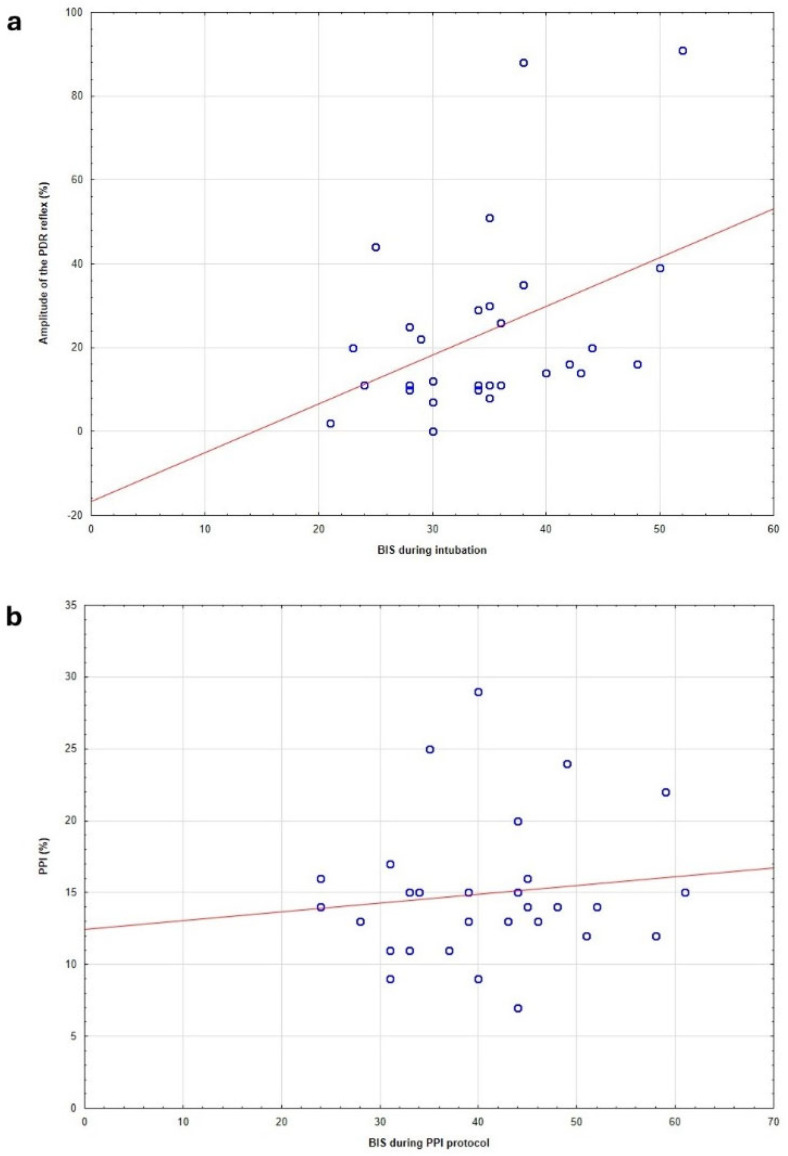
Correlation between bispectral index (BIS) and pupillary responses in the study group. (**a**) Relationship between the BIS and the Pupillary Dilation Reflex (PDR) in patients within group 2 (study group); a statistically significant correlation was observed between these parameters during intubation, indicating that PDR increased with increasing BIS values. (**b**) Relationship between the Pupillary Pain Index (PPI) and BIS in group 2 (study group). No correlation was found between the PPI score and BIS values.

**Figure 5:**
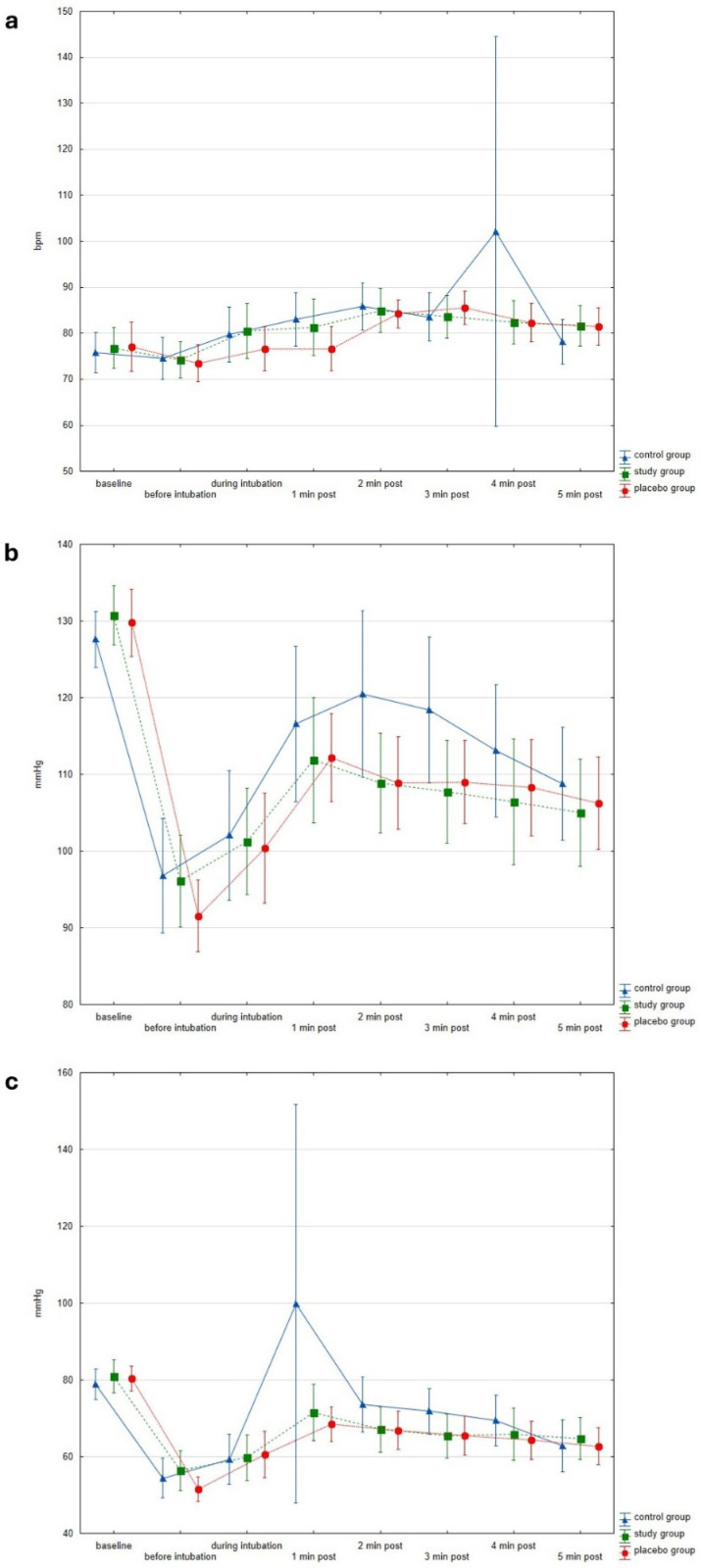
Hemodynamic responses to intubation across study groups. Figure illustrates the hemodynamic changes observed in each group immediately before, during, and at 1, 2, 3, 4, and 5 min post-intubation. (**a**) Compares Heart Rate (HR) values across the groups. The *y*-axis represents the numerical HR. (**b**) Compares Systolic Blood Pressure (SBP) values across the groups. The *y*-axis represents the numerical SBP in mmHg. (**c**) Compares Diastolic Blood Pressure (DBP) values across the groups. The *y*-axis represents the numerical DBP in mmHg.

The magnitude of pupillary dilation during the PRD protocol was significantly influenced by the depth of anesthesia. We observed a positive correlation between PDR and BIS values, indicating that pupillary diameter increased as BIS values increased (signifying a diminished level of anesthesia; Figure 4a) and decreased as BIS values decreased. Conversely, no correlation was found between the PPI score and BIS values (Figure 4b). No statistically significant differences were observed among the three groups for heart rate (HR), systolic blood pressure (SBP), and diastolic blood pressure (DBP) at any measured time point throughout the study protocol (*p* > 0.05). The hemodynamic changes within the two groups are presented in Figure 5a–c.

## 4. Discussion

Accurate assessment of analgesic adequacy during general anesthesia remains a cornerstone of perioperative care. In this context, pupillometry has emerged as a promising technique for monitoring nociceptive reflexes, offering a physiological, real-time measure of the nociception–antinociception balance [7]. While infrared pupillometry has existed for decades, its application for intraoperative nociceptive assessment is a relatively recent development. The advent of automated pupil tracking systems has addressed previous concerns regarding operator dependency and measurement variability, enabling more objective and reproducible assessments of the PDR [10]. Despite this technological progress, the influence of various factors such as opioid administration, age, gender, and concurrent medications on PDR is not fully understood and requires further investigation.

The current study is among the first to specifically investigate PDR during orotracheal intubation, an intense nociceptive stimulus frequently encountered during the induction of anesthesia. Prior studies have examined hemodynamic responses to intubation, including heart rate and blood pressure changes, but these variables are subject to confounding factors such as baseline cardiovascular status and concurrent pharmacologic interventions. For instance, medications like clonidine, dexmedetomidine, esmolol, ketamine, and lidocaine have all been evaluated for their effects on the hemodynamic response to laryngoscopy and intubation [11,12]. However, nociceptive reflexes such as PDR may offer a more sensitive and specific marker of analgesic adequacy, as they directly reflect autonomic responses to noxious stimuli [13].

### 4.1. Pupillary Reflex Physiology and Nociception Monitoring

The pupil size is controlled by the dynamic interplay between the sympathetic and parasympathetic nervous systems, with the parasympathetic system mediating miosis via midbrain pathways and the sympathetic system inducing mydriasis through a polysynaptic arc [5]. Under general anesthesia, the PDR primarily results from parasympathetic inhibition, making it a valuable marker of nociceptive input in anesthetized patients [14]. Our findings are consistent with this physiological model, as pupillary dilation in response to intubation reflects the central autonomic processing of noxious stimuli, even when patients are deeply anesthetized.

Despite the increasing availability of intraoperative nociception monitors, such as the Analgesia Nociception Index (ANI) and the Nociceptive Flexion Reflex (NFR), these devices primarily indicate inadequate analgesia and do not easily differentiate between under- and overdosing of analgesics [3,15].

### 4.2. Lidocaine and Pupillary Response

Our results suggest that intravenous lidocaine administered prior to laryngoscopy can attenuate the PDR, supporting the hypothesis that lidocaine modulates central sensitization and sympathetic activation. Patients receiving lidocaine demonstrated a significantly reduced median PDR (0.33 mm vs. 0.50 mm, *p* = 0.02), indicating decreased nociceptive input during airway manipulation. This finding aligns with prior studies demonstrating that lidocaine blunts hemodynamic and neuroendocrine responses to intubation [16,17,18]. The analgesic properties of lidocaine, both peripherally via sodium channel blockade and centrally via spinal cord desensitization, are well-documented [19]. Our study extends this knowledge by showing that lidocaine’s effects are also detectable through pupillometry, a more direct measure of nociceptive autonomic reflexes.

### 4.3. Correlation with Anesthesia Depth

Another important observation was the positive correlation between PDR values and BIS scores, suggesting that lighter anesthesia (higher BIS) is associated with greater pupillary dilation during noxious stimulation. This finding is consistent with previous reports, notably by Gruenewald et al. [2], who demonstrated that pupillometry reflects the analgesic component of anesthesia and can guide opioid administration intraoperatively. However, the lack of significant differences in PPI scores between the study groups could be explained by the standardized anesthetic depth (BIS ≤ 50), which may have blunted higher cortical processing of nociceptive stimuli. As a result, while PDR measurements captured autonomic nociceptive responses, the PPI scores may not have fully differentiated the groups due to the uniform hypnotic state.

### 4.4. Hemodynamic Parameters and Analgesic Assessment

Interestingly, our study did not identify statistically significant differences in HR, SBP, or DBP between the groups at any point. This finding might reflect the protective effects of fentanyl, lignocaine and propofol, both of which are known to attenuate sympathetic responses [20,21]. Additionally, the tight BIS control likely contributed to hemodynamic stability. It is noteworthy that while hemodynamic markers are often used to infer nociception, they are subject to numerous confounders, including volume status, autonomic tone, and pharmacologic agents [22]. Therefore, our findings highlight the potential of pupillometry as a more sensitive marker for nociceptive events, especially in scenarios where hemodynamic changes are muted or masked.

### 4.5. Clinical Implications

From a clinical perspective, the use of pupillometry during intubation may uncover individual variability in pain sensitivity that conventional monitoring cannot detect. Our data revealed that a higher proportion of patients in the placebo group exhibited “very strong” PDR responses, suggesting greater nociceptive reactivity in the absence of lidocaine. These observations support the concept of individualized nociception monitoring, where objective physiological parameters guide analgesic administration to optimize patient care and potentially improve postoperative outcomes.

Moreover, the findings advocate for the incorporation of multimodal monitoring strategies during anesthesia, combining standard tools like BIS with nociceptive assessments such as PDR. This approach aligns with recent trends in precision medicine, emphasizing tailored therapeutic interventions based on real-time physiological feedback.

### 4.6. Limitations

Despite its promising results, this study has several limitations. The sample size, while adequate for detecting primary outcome differences in PDR, may have been underpowered to capture subtle variations in hemodynamic responses. Additionally, we did not evaluate long-term postoperative pain or opioid consumption, which could provide further insights into the broader benefits of intraoperative nociception monitoring and lidocaine administration. Furthermore, while BIS monitoring is a widely accepted tool for assessing hypnotic depth, it may not comprehensively reflect all aspects of consciousness and pain processing [23]. Future studies could integrate additional neurophysiological markers to complement BIS and pupillometry.

## 5. Conclusions

In conclusion, our study demonstrates that intravenous lidocaine attenuates the Pupillary Dilation Reflex during tracheal intubation, suggesting a significant role in modulating nociceptive input. Pupillometry proves to be a reliable and objective measure of nociceptive responses, even under general anesthesia, and may represent an important addition to intraoperative monitoring protocols. By providing real-time feedback on nociception–antinociception balance, pupillometry may help anesthesiologists individualize analgesic management, potentially improving patient outcomes. Further research is warranted to refine nociception monitoring techniques and assess their impact on postoperative recovery.

## Figures and Tables

**Table 1:** Comparison of pupillary dilation reflex (pdr) parameters between study and control groups. This table summarizes the quantitative and qualitative characteristics of the Pupillary Dilation Reflex (PDR) in both group 1 (control) and group 2 (study). Quantitative data are presented as range, median, and mean ± standard deviation for absolute PDR values (mm) and percentage change (%). No statistically significant differences were found between groups for either measurement (*p* = 0.76 for mm, *p* = 0.80 for % change). The qualitative PDR scale categorizes sensitivity as null, weak, strong, or very strong. The distribution of these categories was similar between the groups (*p* = 0.86). The majority of patients in both groups exhibited a “very strong” PDR, with 43% in the control group and 45% in the study group; PDR—Pupillary Dilation Reflex; *n*—number of patients; *p*—statistical significance (*p*-value).

PDR	Group 1 (Control)				Group 2 (Study)				*p*
	*n* (%)	Range	Median	Mean ± Standard	*n* (%)	Range	Median	Mean ± Standard	
mm	28 (100)	0.08–2.88	0.34	0.56 ± 0.63	29 (100)	0.01–1.91	0.33	0.48 ± 0.46	0.76
%	28 (100)	2.0–140.0	14.0	26.2 ± 30.3	29 (100)	0–91.0	16.0	23.6 ± 21.9	0.80
sensitivity scale									0.86
null	1 (3)				2 (7)				
weak	8 (29)				9 (31)				
strong	7 (25)				5 (17)				
very strong	12 (43)				13 (45)				

**Table 2:** Qualitative and quantitative comparison of pupillary dilation reflex (PDR) between study and placebo groups. This table presents a comparison of the Pupillary Dilation Reflex (PDR) between the study group (group 2) and the placebo group (group 3). A statistically significant difference in pupil dilation was observed between the groups. The median absolute pupil dilation in the study group was 0.33 mm, while in the placebo group it was 0.50 mm (*p* = 0.02). Similarly, the percentage of pupil dilation was significantly lower in the study group, with a median of 16%, compared to 26% in the placebo group (*p* = 0.04). In the qualitative assessment based on the PDR scale, the study group predominantly exhibited high sensitivity to pain, whereas the placebo group demonstrated very high sensitivity to pain. Specifically, 45% of patients in the study group had a “very strong” PDR response, compared to 65.5% in the placebo group. Although the distribution of responses on the PDR scale indicated a trend toward stronger reactions in the placebo group, this difference did not reach statistical significance (*p* = 0.10); *n*—number of patients; *p*—statistical significance (*p*-value).

PDR	Group 2 (Study)				Group 3 (Placebo)				*p*
	*n* (%)	Range	Median	Mean ± Standard	*n* (%)	Range	Median	Mean ± Standard	
mm	29 (100)	0.01–1.91	0.33	0.48 ± 0.46	29 (100)	0.06–2.31	0.50	0.66 ± 0.49	0.02
%	29 (100)	0–91.0	16.0	23.6 ± 21.9	29 (100)	3.0–96.0	26.0	31.9 ± 22.6	0.04
sensitivity scale									0.10
null	2 (7)				1 (3.5)				
weak	9 (31)				2 (7)				
strong	5 (17)				7 (24)				
very strong	13 (45)				19 (65.5)				

**Table 3:** (**a**–**c**) Qualitative comparison of pupillary pain index (PPI) across control, study, and placebo groups. These tables present a qualitative comparison of the Pupillary Pain Index (PPI) across the study cohorts. Specifically, the table illustrates the qualitative scale of the PPI by comparing: (**a**) group 1 (control) versus group 3 (placebo), (**b**) group 1 (control) versus group 2 (study) and (**c**) group 2 (study) versus group 3 (placebo); *n*: number of patients; *p*: statistical significance.

(**a**)							
	**Control Group (*n* = 28)**			**Study Group (*n* = 29)**			
**PPI Variation**	**Range**	**Median**	**Mean ± Standard**	**Range**	**Median**	**Mean ± Standard**	***p***
PPI scale	2.0–9.0	6.0	5.6 ± 2.4	2.0–9.0	6.0	5.4 ± 2.5	0.74
mm	0.13–0.68	0.3	0.3 ± 0.14	0.14–0.52	0.28	0.30 ± 0.09	0.45
%	7.0–30.0	15.0	15.8 ± 5.9	7.0–29.0	14.0	15.0 ± 4.9	0.68
(**b**)							
	**Study Group (*n* = 29)**			**Placebo Group (*n* = 29)**			
**PPI Variation**	**Range**	**Median**	**Mean ± Standard**	**Range**	**Median**	**Mean ± Standard**	***p***
PPI scale	2.0–9.0	6.0	5.4 ± 2.5	2.0–9.0	5.0	5.3 ± 2.5	0.90
mm	0.14–0.52	0.28	0.3 ± 0.09	0.14–0.82	0.31	0.32 ± 0.15	0.84
%	7.0–29.0	14.0	15.0 ± 4.9	7.0–40.0	14.0	15.2 ± 6.6	0.92
(**c**)							
	**Control Group (*n* = 28)**			**Placebo Group (*n* = 29)**			
**PPI Variation**	**Range**	**Median**	**Mean ± Standard**	**Range**	**Median**	**Mean ± Standard**	***p***
PPI scale	2.0–9.0	6.0	5.6 ± 2.4	2.0–9.0	5.0	5.3 ± 2.5	0.67
mm	0.13–0.68	0.3	0.3 ± 0.14	0.14–0.82	0.31	0.32 ± 0.15	0.74
%	7.0–30.0	15.0	15.8 ± 5.9	7.0–40.0	14.0	15.2 ± 6.6	0.64

## Data Availability

The trial was registered at ClinicalTrials.gov (ID: NCT04000126).

## Abbreviations

The following abbreviations are used in this manuscript:

PDR	pupillary reflex dilation
PPI	Pupillary Pain Index
BIS	Bispectral Index
ASA	American Society of Anesthesiologists
ECG	electrocardiography
NIBP	non-invasive blood pressure
TOF	Train-of-Four
SBP	systolic blood pressure
DBP	diastolic blood pressure
HR	heart rate
